# Detection of 5 Kinds of Genes Related to Plasmid-Mediated Quinolone Resistance in Four Species of Nonfermenting Bacteria with 2 Drug Resistant Phenotypes

**DOI:** 10.1155/2020/3948719

**Published:** 2020-04-09

**Authors:** De-Song Ming, Qing-Qing Chen, Xiao-Ting Chen

**Affiliations:** Department of Clinical Laboratory, The First Hospital of Quanzhou Affiliated to Fujian Medical University, Quanzhou 362000, China

## Abstract

**Objective:**

This study aimed to detect 5 kinds of genes related to plasmid-mediated quinolone resistance in four species of nonfermenting bacteria with 2 drug resistance phenotypes (multidrug resistance and pandrug resistance), which were *Acinetobacter baumannii* (Ab), *Pseudomonas aeruginosa* (Pa), *Stenotrophomonas maltophilia* (Sm), and *Elizabethkingia meningoseptica* (Em).

**Methods:**

The Phoenix NMIC/ID-109 panel and API 20NE panel were applied to 19 isolated strains, including 6 Ab strains (2 strains with multidrug resistance and 4 strains with pandrug resistance), 6 Pa strains (3 strains with multidrug resistance and 3 strains with pandrug resistance), 4 Sm strains (2 strains with multidrug resistance and 2 strains with pandrug resistance), and 3 Cm strains (2 strains with multidrug resistance and 1 strain with pandrug resistance). After strain identification and drug susceptibility test, PCR was applied to detect 5 genes related to plasmid-mediated quinolone resistance. The genes detected were quinolone resistance A (*qnrA*), aminoglycoside acetyltransferase ciprofloxacin resistance variant, *acc(6′)-Ib-cr*, and 3 integrons (*intI*1, *intI*2, and *intI*3). The amplified products were analyzed by 1% agarose gel electrophoresis and sequenced. Sequence alignment was carried out using the bioinformatics technique.

**Results:**

Of 19 strains tested, 8 strains carried *acc(6′)-Ib-cr* and 6 of them were of pandrug resistance phenotype (3 Ab strains, 2 Pa strains, and 1 Sm strain). The carrying rate of *acc(6′)-Ib-cr* was 60.0% for strains of pandrug resistance (6/10). Two strains were of multidrug resistance (1 Ab strain and 1 Pa strain), and the carrying rate of *acc(6′)-Ib-cr* was 22.0% (2/9). The carrying rate was significantly different between strains of multidrug resistance and pandrug resistance (*P* < 0.05). The class 1 integron was detected in 11 strains, among which 6 strains were of pandrug resistance (3 Ab strains, 2 Pa strains, and 1 Sm strain). The carrying rate of the class 1 integron was 60.0% (6/10). Five strains were of multidrug resistance (3 Pa strains, 1 Ab strain, and 1 Em strain), and the carrying rate was 55.6% (5/9). The carrying rate of the class 1 integron was not significantly different between strains of multidrug resistance and pandrug resistance (*P* > 0.05). Both *acc(6′)-Ib-cr* and *intI*1 were detected in 6 strains, which were negative for *qnrA*, *intI*2, and *intI*3.

**Conclusion:**

Quinolone resistance of isolated strains was related to *acc(6′)-Ib-cr* and *intI*1 but not to *qnrA*, *intI*2, or *intI*3. The carrying rate of *acc(6′)-Ib-cr* among the strains of pandrug resistance was much higher than that among the strains of multidrug resistance. But, the strains of two drug resistant phenotypes were not significantly different in the carrying rate of *intI*1. The detection rates of the two genes were high and similar in Ab and Pa strains. 1 Em strain carried the class 1 integron.

## 1. Introduction

*Acinetobacter baumannii* (Ab), *Pseudomonas aeruginosa* (Pa), and *Stenotrophomonas maltophilia* (Sm) are common nonfermenting bacteria in clinic [1–3], while *Elizabethkingia meningoseptica* (Em) is relatively rare [4]. Mutations in these bacteria are largely attributed to overuse and misuse of antibiotics, and drug resistance of these bacteria has become a crisis [5]. Many studies are devoted to the mechanism of quinolone resistance in Ab, Pa, and Sm, but only a few studies are published regarding quinolone resistance in Cm or genes related to plasmid-mediated quinolone resistance and their relationship with different drug resistance phenotypes [6–9]. We detected 5 genes related to plasmid-mediated quinolone resistance in 19 isolated strains (2 Ab strains with multidrug resistance, 4 Ab strains with pandrug resistance, 3 Pa strains with multidrug resistance, 3 Pa strains with pandrug resistance, 2 Sm strains with multidrug resistance, 2 Sm strains with pandrug resistance, 2 Cm strains with multidrug resistance, and 1 Cm strain with pandrug resistance). The genes detected were quinolone resistance A (*qnrA*), aminoglycoside acetyltransferase ciprofloxacin resistance variant, *acc(6′)-Ib-cr*, and 3 kinds of integrons (*intI*1, *intI*2, and *intI*3) by using PCR with homology analysis. The purpose of this study was to understand the mechanism of quinolone resistance and its relationship with drug resistance phenotype in clinical strains of Ab, Pa, Sm, and Em.

## 2. Materials and Methods

### 2.1. Strains

All 19 strains (2 Ab strains with multidrug resistance, 4 Ab strains with pandrug resistance, 3 Pa strains with multidrug resistance, 3 Pa strains with pandrug resistance, 4 Sm strains, and 3 Cm strains) were clinically isolated and preserved at our hospital. Strain No., identification code, and time of isolation are shown in Table 1.

### 2.2. Reagents

Phoenix NMIC/ID-109 panel (BD Corporation, BD), M-H agar medium (Oxoid, UK), and API 20NE panel/PSE5.0 susceptibility strip (bioMérieux, France) were used.

### 2.3. Strain Identification

Bacterial culture was prepared conventionally. The colonies were purified and subjected to the O/F test and oxidase test. The above procedures were performed using a Phoenix NMIC/ID-109 panel, API 20NE panel, and PSE5.0 susceptibility strip.

### 2.4. Drug Susceptibility Test

MIC was performed using an API 20NE panel, PSE5.0 susceptibility strip, and/or Phoenix NMIC/ID-109 panel according to NCCLS guidelines [10]. For quality control, Pa strain ATCC27853 was used to test the reagents every week.

### 2.5. Detection of Genes Related to Plasmid-mediated Quinolone Resistance

PCR was applied to 5 genes related to plasmid-mediated quinolone resistance (*acc(6′)-Ib-cr*, *qnrA,* and 3 integrons (*intI*1, *intI*2, and *intI*3). The primer sequences and PCR procedures were described in the literature [11]. The amplified products were analyzed by 1% agarose gel electrophoresis.

### 2.6. Sequencing and Alignment

The amplified genes were purified and sequenced. Homologous sequences were searched using BLAST program in GenBank. The accession number of homologous sequence for *acc(6′)-Ib-cr* was EF375621.

## 3. Results

### 3.1. Drug Resistance

All 19 strains were tested by MICs for drug susceptibility using the Phoenix NMIC/ID-109 panel and PSE5.0 susceptibility strip. Ten strains were of pandrug resistance phenotype and 9 strains were of multidrug resistance phenotype (Table 1).

### 3.2. Carrying Rates of 5 Detected Genes in Two Drug Resistance Phenotypes

**Figure 1:**
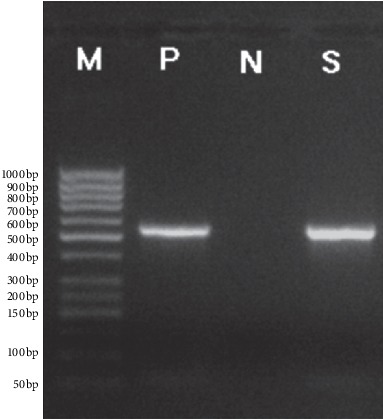
Electrophogram of *aac(6′)-Ib-Cr* genes by PCR. S, positive sample; N, negative control; P, positive control; M, DNA marker.

**Figure 2:**
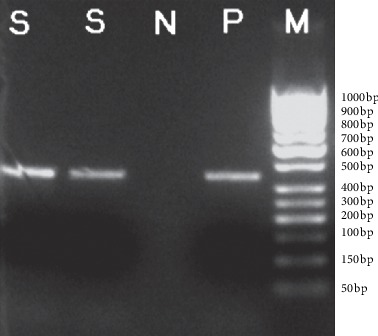
Electrophogram of *intI*1 genes by PCR. S, positive sample; N, negative control; P, positive control; M, DNA marker.

Of 19 strains detected by PCR, 8 strains carried *acc(6′)-Ib-cr* and 6 of them were of pandrug resistance phenotype (3 Ab strains, 2 Pa strains, and 1 Sm strain). The carrying rate of *acc(6′)-Ib-cr* was 60.0% for strains of pandrug resistance (6/10). Two strains were of multidrug resistance (1 Ab strain and 1 Pa strain), and they were only negative in the ciprofloxacin susceptibility test. The carrying rate of *acc(6′)-Ib-cr* was 22.0% (2/9). The carrying rate was significantly different between strains of multidrug resistance and pandrug resistance (*P* < 0.05). The class 1 integron was detected in 11 strains, among which 6 strains were of pandrug resistance (3 Ab strains, 2 Pa strains, and 1 Sm strain). The carrying rate of the class 1 integron was 60.0% (6/10). Five strains were of multidrug resistance (3 Pa strains, 1 Ab strain, and 1 Cm strain), and they were only negative in the ciprofloxacin susceptibility test. The carrying rate was 55.6% (5/9). The carrying rate of the class 1 integron was not significantly different between strains of multidrug resistance and pandrug resistance (*P* > 0.05). Both *acc(6′)-Ib-cr* and *intI*1 were detected in 6 strains, which were of pandrug resistance (3 Ab strains, 1 Pa strain, and 2 multidrug resistance strains). Five strains were found to carry only *intI*1, and 2 strains were found to carry only *acc(6′)-Ib-cr*. They were negative for *qnrA*, *intI*2, and *intI*3 (Table 1, Figures 1 and 2).

### 3.3. Distribution of Drug Resistance-Related Genes in 4 Bacterial Species

Of 6 Ab strains, 4 strains carried both *acc(6′)-Ib-cr* and *intI*1 (3 strains with pandrug resistance and 1 strain with multidrug resistance) and none carried only one drug resistance gene. Of 6 Pa strains, 3 strains carried *acc(6′)-Ib-cr*, 5 strains *intI*1, and 2 strains both two genes. No. 5 Sm strain was positive for *acc(6′)-Ib-cr*, and No. 7 Sm strain was positive for *intI1*. No. 19 Cm strain, which was of multidrug resistance, carried *intI*1. Other Cm strains were negative for *intI*1 and positive for *qnrA*, *intI*2, and *intI*3.

### 3.4. Homology Analysis

**Figure 3:**
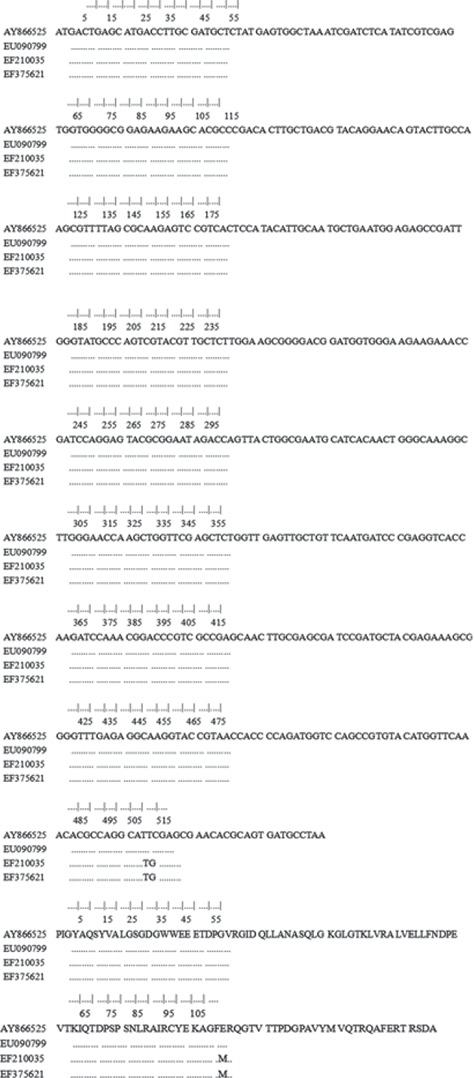
Alignment with sequence EF210035.

The amplified product of *acc(6′)-Ib-cr* was sequenced, and it contained 519 nucleic acids. By alignment with sequence (accession number EF375621) in GenBank, the identity was above 99% without sense mutation. This sequence was verified as *acc(6′)-Ib-cr*. The accession number of *acc(6′)-Ib-cr* from No. 5 Sm strain was EF210035. DNA and amino acid sequence alignment was two *acc(6′)-Ib* sequences (AY866525 and EU090799) and one *acc(6′)-Ib-cr* sequence (EF375621) (Figure 3).

## 4. Discussion

Chromosomally mediated mechanisms mainly underlie quinolone resistance, such as change of the target site induced by drugs, decreased permeability of outer membrane porin, and active pumping of efflux pump [12]. Drug resistance in Pa may be mediated by plasmids (involving 4 genes, *qnr* (protection protein), *qepA*, *oqxAB* (efflux pump), and a*cc(6′)-Ib-cr* (quinolone-modifying enzyme)) [8]. As the issue of drug resistance is intensifying, the pandrug resistance phenotype has emerged [5]. Genes related to plasmid-mediated quinolone resistance may be engaged in dissemination of genes related to drug resistance mediated by other plasmids [6–9]. Therefore, different genes are involved in quinolone resistance for different drug resistance phenotypes (multidrug or pandrug resistance).

qnrA belongs to the qnr families that protect DNA gyrase from quinolones; *qnrB* and *qnrA* are the main genes related to plasmid-mediated quinolone resistance carried by bacteria [6]. However, qnrA was not detected in any of the 19 strains, suggesting that this gene was not related to quinolone resistance in the 4 species of strains tested. Touati et al. [13] and Güler and Eraç [14] also reported that *qnr* is not the main mechanism of quinolone resistance in Ab strains.

*acc(6′)-Ib-cr* participates in plasmid-mediated quinolone resistance, encoding for the *acc(6′)-Ib* enzyme that leads to resistance to norfloxacin and ciprofloxacin [6–9]. Of 19 strains tested, 8 genes carried this gene and 6 of them showed pandrug resistance phenotype. The remaining 2 strains were of multidrug resistance phenotype, and they were only negative in the ciprofloxacin susceptibility test. This means *acc(6′)-Ib-cr* is involved in the quinolone resistance of Ab, Pa, Sm, and Cm. The carrying rate of this gene was higher for the pandrug resistance phenotype than for the multidrug resistance phenotype. The detection rate of *acc(6′)-Ib-cr* was higher and similar in Ab and Pa strains.

Integrons are associated with plasmid-mediated quinolone resistance, and class 1, 2, and 3 integrons are the most common, the genetic markers being *intI*1, *intI*2, and *intI*3, respectively. Of 19 strains, *intI*1 was detected in 11 strains in all species of Ab, Pa, Sm, and Em, among which 6 strains were of pandrug resistance phenotype and 5 strains of multidrug resistance phenotype. Thus, the class 1 integron was related to *intI*1, but no significant difference was found in the carrying rate between the pandrug resistance phenotype and multidrug resistance phenotype (*P* > 0.05). Class 2 and 3 integrons were not detected in any of the strains, which agreed with previous reports [15–18]. Therefore, class 2 and 3 integrons were not related to quinolone resistance in the tested strains. *intI*1 had a high and similar detection rate in Ab and Pa strains. Few reports were on the class 1 integron in EM [19, 20]; in this paper, 1 Em strain carried the class 1 integron.

Both *acc(6′)-Ib-cr* and *intI*1 were detected in 6 strains (4 pandrug resistance strains (3 Ab strains and 1 Pa strain) and 2 multidrug resistance strains). Five strains carried only *intI*1, and 2 strains carried only *acc(6′)-Ib-cr*. The strains carrying both *acc(6′)-Ib-cr* and *intI*1 mostly belonged to pandrug resistance phenotype. Moreover, it is proved that *acc(6′)-Ib-cr* exists in *intI*1 [6–9].

To conclude, the mechanism of plasmid-mediated quinolone resistance is mainly related to *acc(6′)-Ib-cr* and *intI*1, which have high and similar detection rates in Ab and Pa strains, but not to *qnrA*, *intI*2, or *intI*3. The carrying rate of *acc(6′)-Ib-cr* was higher in the pandrug resistance phenotype than in the multidrug resistance phenotype. However, the two phenotypes did not differ significantly in the carrying rate of *intI*1, and *acc(6′)-Ib-cr* exists in *intI*1. 1 Em strain carried the class 1 integron. Also, further studies are needed to investigate the resistance mechanisms, ESBLs, MBLs, OXA-type carbapenemases, and aminoglycoside resistance determinants in the 19 strains.

## Figures and Tables

**Table 1:** Basic data and results of AST and PCR of 19 clinical strains.

No.	No. of sample	Type of specimens	Germ name	API ID card	BD ID%	Isolated time	Results of AST	Results of PCR				
								*qnrA*	*aac(6′)-Ib-Cr*	*intI*1	*intI*2	*intI*3
1	91112-2	Sputum	Ab	204042	90	2006-9-14	PR	−	−	−	−	−
2	90911	Sputum	Ab	204042	99	2006-9-12	PR	−	+	+	−	−
3	82826-2	Sputum	Ab	204042	90	2006-8-26	PR	−	+	+	−	−
4	101907	Sputum	Ab	NT	99	2006-10-19	PR	−	+	+	−	−
5	1370	Blood	Ab	204051	99	2003-8-28	MR/CIP R	−	+	+	−	−
6	1532	Sputum	Ab	204042	96	2003-9-5	MR/CIP S	−	−	−	−	−
7	B736	Blood	Pa	1054555	99	2003-7-4	MR/CIP R	−	−	+	−	−
8	92010	Sputum	Pa	1154575	99	2006-9-20	MR/CIP R	−	−	+	−	−
9	90708	Sputum	Pa	1050075	95	2006-9-7	MR/CIP R	−	+	+	−	−
10	102021	Sputum	Pa	NT	99	2006-10-20	PR	−	+	−	−	−
11	50407-1	Wound pus	Pa	NT	99	2006-5-4	PR	−	+	+	−	−
12	90821	Sputum	Pa	1154475	99	2006-9-8	PR	−	−	+	−	−
13	52616	Sputum	Sm	1472344	99	2006/5/26	PR	−	+	−	−	−
14	92930-2	Sputum	Sm	1472341	99	2006/10/2	PR	−	−	+	−	−
15	1689	Blood	Sm	1472345	95	2003/8/18	MR	−	−	−	−	−
16	51108	Sputum	Sm	1432355	99	2006/5/11	MR	−	−	−	−	−
17	61424	Sputum	Em	2476004	99	2006-6-14	PR	−	−	−	−	−
18	287	Blood	Em	1610204	NT	2004-3-12	MR/LEV R	−	−	−	−	−
19	2700	Sputum	Em	2442244	NT	2004-12-9	MR/LEV R	−	−	+	−	−

Ab, *Acinetobacter baumannii*; Pa, *Pseudomonas aeruginosa*; Sm, *Stenotrophomonas maltophilia*; Em, *Elizabethkingia meningoseptica*; NT, undetected; PR, pandrug resistant; MR, MDR; *R*, resistance; *S*, sensitive. CIP, ciprofloxacin; LEV, Levofloxacin; “+,” positive; and “−,” negative.

## Data Availability

The data used to support the findings of this study are available from the corresponding author upon request.
